# Consistency of Subcortical Osseous Structures in Femoral Cruciate Ligament Attachment Sites: A Combined 3D-CT and Histological Anatomical Study

**DOI:** 10.3390/jpm16070352

**Published:** 2026-06-29

**Authors:** Johannes Moritz Mittendorfer, Zehra Duezguen, Lukas Horak, Andreas Gahleitner, Elisabeth Marlene Mandler, Lena Hirtler

**Affiliations:** 1 Division of Anatomy, Center for Anatomy and Cell Biology, Medical University of Vienna, 1090 Vienna, Austria; johannes.mittendorfer@meduniwien.ac.at (J.M.M.); zehra.duezguen@meduniwien.ac.at (Z.D.); elisabeth.mandler@meduniwien.ac.at (E.M.M.); 2 Division of Neuroradiology and Muskuloskeletal Radiology, Department of Radiology and Image-Guided Therapy, Medical University of Vienna, 1090 Vienna, Austria

**Keywords:** anterior cruciate ligament, posterior cruciate ligament, enthesis, intercondylar ridge, resident’s ridge, bifurcate ridge, computed tomography, histology, precision orthopedics, patient-specific planning

## Abstract

**Background/Objectives**: Accurate identification of the functional femoral attachment of the anterior (ACL) and posterior cruciate ligament (PCL) is essential for anatomic reconstruction and for patient-specific femoral tunnel planning, yet correct intraoperative localization remains inconsistent. This anatomical study investigated whether subcortical bone features provide evidence of functional cruciate ligament attachment and whether surrounding osseous ridges are reliable landmarks. **Methods**: Computed tomography (CT) scans of 20 paired, fresh-frozen distal femora (10 body donors) were processed using 3D volume rendering to visualize the intercondylar fossa walls and to assess the presence of four characteristic ridges: lateral intercondylar ridge (LIR), lateral bifurcate ridge (LBR), medial intercondylar ridge (MIR) and medial bifurcate ridge (MBR). In addition, thin-ground section histology of the femoral insertion sites was performed to characterize insertion morphology. **Results**: Histology demonstrated distinct direct and indirect insertions for both ligaments; the direct insertion exhibited a characteristic four-layer transition (ligament, non-calcified fibrocartilage, calcified fibrocartilage, bone), whereas the indirect insertion showed collagen fibers attaching directly to bone. The LIR and MIR were present in 85% and 80% of specimens, respectively, while the LBR and MBR were less frequent (LBR 25%, MBR 10%). No significant associations were found between ridge presence and age, sex or laterality. **Conclusions**: These findings support the direct insertion as the functional cruciate attachment and suggest that the LIR and MIR—due to their consistent occurrence and location at the rim of the direct insertion—are the most useful bony landmarks for individualized femoral tunnel orientation, whereas bifurcate ridges should be considered adjunctive when present. The observed variability provides a rationale to stratify cases requiring adjunct imaging or navigation.

## 1. Introduction

Anterior cruciate ligament (ACL) rupture is a frequent cause of symptomatic knee instability and is commonly associated with secondary meniscal and chondral injury, motivating surgical reconstruction in appropriately selected patients [1]. Contemporary ACL reconstruction (ACLR) seeks to restore near-native knee kinematics by reproducing the native insertional anatomy and fiber orientation; among modifiable technical factors, femoral tunnel position is consistently emphasized as one of the dominant determinants of graft function because small deviations can substantially affect graft length-change behavior, graft bending angle, and in vivo graft loading patterns [2] and may translate into residual instability or graft failure [3]. Accordingly, malpositioned tunnels have been linked to inferior outcomes and higher revision risk, reinforcing that “anatomic” positioning is clinically consequential [4,5].

Within the broader paradigm of personalized medicine, orthopedic surgery increasingly aims to tailor operative strategy to the individual patient’s anatomy, biomechanics, and risk profile, supported by advanced imaging and digital planning [6,7,8]. In ACL reconstruction, patient-specific approaches—including CT/MRI-based 3D modeling and patient-specific instrumentation—have been proposed to reduce tunnel-placement outliers in the setting of limited arthroscopic visibility and anatomical variance [9].

Because intraoperative footprint localization is challenging, objective postoperative assessment methods have become integral to quality control and research, and three-dimensional computed tomography (3D-CT) is widely used to evaluate femoral tunnel aperture position and quantify deviations from intended targets [2]. Complementary imaging work has also explored three-dimensional MRI-based guidance of tibial and femoral tunnel position under experimental conditions [10]. Standardized coordinate systems—most prominently the quadrant method—enable reproducible reporting of femoral tunnel position [11], and contemporary adaptations aim to improve usability and reliability across imaging modalities [12].

Building on these principles, fluoroscopy-guided placement has been proposed to improve precision, particularly among lower-volume surgeons or in settings where arthroscopic landmark identification is uncertain [13]. Nevertheless, purely image- or measurement-based approaches can increase complexity, and the fundamental intraoperative problem persists: arthroscopic localization of the native femoral footprint is constrained by inter-individual anatomic variability, inconsistent ridge visibility, and changes related to chronic ACL deficiency and notch remodeling [14,15].

Accordingly, substantial attention has focused on osseous landmarks of the intercondylar wall, particularly the lateral intercondylar ridge (LIR, “resident’s ridge”) and the lateral bifurcate ridge (LBR), which are described as borders or internal dividers of the ACL femoral footprint [15,16,17]. However, controlled three-dimensional model studies indicate that even trained surgeons may inconsistently identify these ridges and the intended tunnel target, highlighting the need for more objective linkage between visible anatomy and the true functional attachment [16,18]. Adding further complexity, the macroscopic “footprint” is not invariant: anatomical evidence indicates that the morphology of the femoral ACL footprint changes with aging—from a larger semicircular configuration to a smaller ribbon-like configuration—suggesting that fixed assumptions about footprint shape or landmark relationships may be inappropriate across patient populations [19]. In parallel, alternative arthroscopic landmark concepts have been developed to improve reproducibility, including cartilage-based references such as the apex of the deep cartilage (ADC) and posterior femoral cartilage reference points, with studies supporting their utility for tunnel evaluation or intraoperative positioning [19,20] and related ADC-based validation/positioning work [21,22].

Beyond gross arthroscopic morphology, microanatomic and histologic studies distinguish a dense “direct” femoral insertion—often characterized by a fibrocartilaginous transition zone—from a broader “indirect” extension, supporting the concept that a functionally dominant attachment region may not coincide exactly with visually defined footprint boundaries [23]. Emerging mechanobiologic work further reinforces that attachment regions are specialized for force transfer: ultrastructural analyses demonstrate region-specific mineralization interface characteristics across human ACL entheses consistent with adaptation to local mechanical demands [24], and complementary translational studies describe alterations in mineralized tissue state at the femoral ACL enthesis in the setting of ACL failure [25]. Consistent with the premise that local loading drives bone adaptation, CT-based mapping of subcortical bone density along the lateral intercondylar wall has identified high-density regions whose centers approximate reported anatomic footprint centers, suggesting that subcortical bone characteristics may provide objective information about functionally loaded attachment [23] and is aligned with broader imaging-based attachment-localization approaches [26]. Importantly, ridge-based orientation is not limited to the ACL: ridge-like landmarks have also been described for the posterior cruciate ligament (PCL) femoral attachment, and recent anatomic, histologic, and biomechanical work continues to refine how distinct fiber regions contribute to function [27,28,29]. Finally, experimental enthesis mechanics studies in animal models provide complementary evidence that insertion-site deformation and attachment angle are load-dependent, supporting the rationale that microstructure and bone adaptation should be considered when defining “functional” attachments [30].

Despite the clinical use of intercondylar ridges for femoral tunnel orientation, it remains unclear which CT-visible osseous landmarks are consistently present and whether these landmarks correspond to histologically defined direct insertion zones of the ACL and PCL. This uncertainty is relevant because reliance on variably expressed or inconsistently visible landmarks may contribute to variability in anatomical tunnel localization. Therefore, this study combined standardized 3D-CT assessment with histological evaluation to determine the prevalence and reproducibility of femoral intercondylar ridges and to explore their relationship to direct and indirect cruciate ligament insertion morphology.

Therefore, the aims of this study were (i) to quantify the prevalence and interobserver reliability of key osseous landmarks of the femoral intercondylar fossa—specifically the lateral intercondylar ridge (LIR), lateral bifurcate ridge (LBR), medial intercondylar ridge (MIR), and medial bifurcate ridge (MBR)—using standardized three-dimensional computed tomography (3D-CT) reorientation and volume-rendered assessment; and (ii) to characterize the microanatomy of the femoral ACL and PCL insertion sites by histology, with particular emphasis on distinguishing direct from indirect insertion zones. We hypothesized that the intercondylar ridges (LIR/MIR) would be detected more frequently and with higher interobserver agreement than the bifurcate ridges (LBR/MBR). Because the histological analysis was descriptive and not based on quantitative CT–histology co-registration, the relationship between ridge-like elevations and direct insertion zones was evaluated exploratively.

## 2. Materials and Methods

### 2.1. Preparation

Twenty fresh-frozen femurs from 10 body donors (left: n = 10; right: n = 10) originating from the Centre for Anatomy and Cell Biology, Medical University of Vienna, Austria were examined. All donors gave informed consent for their bodies to be used postmortem for scientific purposes and teaching. The study was approved by the Ethics Committee of the Medical University of Vienna (EK Nr: 1540/2019). The average donor age was 75.3 (63–92) years; 70% of the donors were female with an average age of 73.7 (63–92) years and 30% were male with an average age of 79.0 (78–81) years.

CT scans were first obtained from fresh-frozen body donors. Following imaging, the femora (n = 20) were prepared for histological evaluation by removal of the joint capsule, muscles, and other soft surrounding tissues. Subsequently, the ACL and PCL were transected. Specimens were stored at −20 °C and after standardized thawing at 4 °C for 48 h fixation was performed immediately. Exclusion criteria comprise previous knee surgery, advanced osteoarthritis, traumatic bone defects, and macroscopic damage to the femoral insertion sites. The specimens did not meet any exclusion criteria.

### 2.2. CT Images Analysis

Computed tomography (CT) was performed (Siemens SOMATOM Definition AS; 120 kV, 140 mA; slice thickness of 0.6 mm) (Siemens Healthineers, Erlangen, Germany), and 3D volume-rendered surface models were generated using RadiAnt DICOM Viewer (v2021.1; Medixant, Poznan, Poland).

**Figure 1:**
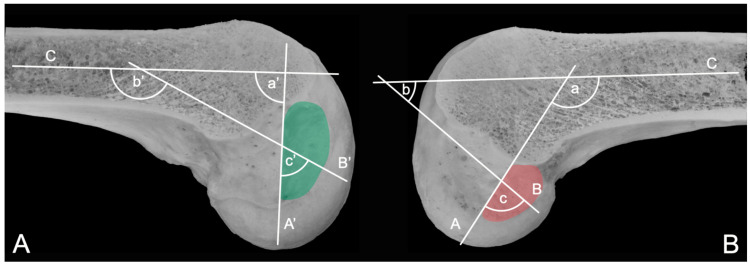
Illustration of all measurements performed. (**A**) Medial wall of the intercondylar notch. Green area signifies the femoral attachment area of the posterior cruciate ligament. A′= axis of the medial intercondylar ridge, B′ = axis of the medial bifurcate ridge, C = longitudinal axis of the femur, a′ = angle between A′ and C, b′ = angle between B′ and C, c′ = angle between A′ and B′. (**B**) Lateral wall of the intercondylar notch. Red area signifies the femoral attachment area of the anterior cruciate ligament. A= axis of the lateral intercondylar ridge, B = axis of the lateral bifurcate ridge, C = longitudinal axis of the femur, a = angle between A and C, b = angle between B and C, c = angle between A and B.

To enable standardized assessment of intercondylar fossa morphology, models were sectioned in the sagittal plane along the Blumensaat line and reoriented to obtain oblique-axial views parallel to the lateral and medial notch walls. Osseous landmarks were evaluated for presence and orientation, focusing on the LIR, LBR, MIR, and MBR (Figure 1). A ridge was scored as present only when a discrete linear osseous elevation could be identified on the corresponding intercondylar wall and followed in the expected anatomical orientation. Broad degenerative irregularities, diffuse cortical unevenness, or osteophyte-like changes without a discrete linear course were not classified as ridges. Both observers were blinded to donor information, specimen side, and histological findings during CT assessment. Before formal evaluation, both observers were trained using a standardized reference dataset illustrating typical examples of the assessed ridges. Uncertain cases were initially recorded independently by both observers and subsequently resolved by consensus after interobserver agreement had been calculated.

Angular measurements were included to characterize spatial orientation of ridges (see Figure 1, Table A1). Measurements were performed relative to the femoral long axis and between ridge axes using ImageJ (version 1.53; NIH, Bethesda, MD, USA).

### 2.3. Histological Analysis

The intercondylar fossa was separated from the diaphysis using a diamond saw (EXAKT^®^, Norderstedt, Germany). Bone blocks (2 × 3 cm each) centered on the femoral ACL and PCL insertion sites were excised, dehydrated in a graded ethanol series, infiltrated with methyl methacrylate (MMA) and embedded in synthetic resin. After polymerization, the sample blocks were sectioned at the ligament insertion site. Section orientation was standardized relative to the intercondylar roof to ensure comparability across specimens. Sections were mounted on coverslips using cyanoacrylate (3M™ Scotch-Weld™ Instant Adhesive CA8, Maplewood, MN, USA) and processed using a diamond angle grinder (cut-grinder primus, Walter Messner GmbH, Hamburg, Germany). Initial slides with a thickness of 250 μm were thinned out to a final thickness of 80–100 μm (EXAKT 400 CS grinder, Norderstedt, Germany). Giemsa staining was performed, followed by light microscopic evaluation (Eclipse Ci-L, Nikon Corporation, Tokyo, Japan) with digital documentation (Nikon Z7, Nikon Corporation). Histological findings were analyzed descriptively for ACL and PCL femoral insertions. The histological evaluation was descriptive. The presence and spatial relationship of ridge-like elevations to direct and indirect insertion zones were recorded qualitatively; no quantitative morphometric co-registration between CT and histological sections was performed. The CT and histological findings were therefore compared descriptively. No specimen-specific quantitative CT–histology co-registration, spatial overlay, or distance-based analysis was performed. Consequently, the relationship between CT-visible ridges and histological insertion morphology was interpreted as an anatomical association rather than direct quantitative correspondence.

### 2.4. Statistics

Statistical analysis was conducted using IBM SPSS^®^ for Mac (Version 28). Ridge presence was assessed by two blinded observers. Both observers were blinded to donor information and were independently trained prior to analysis using a standardized reference dataset. Interrater reliability was assessed prior to consensus evaluation using Landis and Koch’s (1977) [31] interpretation of Cohen’s kappa (κ). Accordingly, evaluation was based on conventional benchmarks (e.g., κ < 0.20 = poor, 0.21–0.40 = fair, 0.41–0.60 = moderate, 0.61–0.80 = substantial, and 0.81–1.00 = almost perfect agreement).

Descriptive statistics were calculated for all variables. Depending on distributional assumptions (Shapiro–Wilk), group comparisons were performed using a two-tailed Student’s *t*-test or Mann–Whitney U, and categorical variables were analyzed using Chi-square tests. Correlations were assessed using Spearman’s correlation coefficient. Statistical significance was set at *p* < 0.05.

Because of the exploratory anatomical design and limited availability of paired fresh-frozen specimens, no formal sample size calculation was performed. The study was designed to generate anatomical and methodological observations rather than confirmatory statistical evidence. Ridge prevalence was reported as n/N, percentage, and 95% confidence intervals. Group differences were reported as absolute percentage-point differences with 95% confidence intervals. Because left and right femora originated from the same donors, the data have a paired donor structure. Owing to the small number of donors and sparse categorical cells, formal multilevel or clustered regression modeling was not performed. Consequently, subgroup comparisons by sex and side should be interpreted as exploratory only, and *p*-values and confidence intervals may overestimate precision if within-donor correlation is present. Categorical comparisons were interpreted cautiously because of small cell counts. *p*-values are presented as exploratory values based on the original analysis and should not be interpreted confirmatorily.

## 3. Results

### 3.1. Analysis of CT Data

The MIR was identified in 16/20 specimens (80%), and the LIR in 17/20 specimens (85%). In contrast, the medial bifurcate ridge (MBR) and lateral bifurcate ridge (LBR) were less frequently observed, occurring in 2/20 (10%) and 5/20 specimens (25%), respectively (Table 1).

Exploratory analyses did not indicate clear sex- or side-related patterns in ridge presence. However, subgroup sizes were small and confidence intervals were wide; therefore, the absence of statistically significant differences should not be interpreted as evidence of no association. Although the LIR was more frequently identified on the right side (10/10) compared to the left (7/10), this difference did not reach statistical significance (χ^2^ = 3.529, *p* = 0.060). Likewise, no consistent relationship was found between donor age and ridge occurrence.

Interobserver agreement for ridge identification was evaluated using Cohen’s kappa coefficient (Table 1). Cohen’s kappa values ranged from 0.773 to 0.894 (*p* < 0.001).

Due to limited sub-sample sizes, only the angle between the femoral axis and the medial intercondylar ridge (aF_MIR) and the femoral axis and the lateral intercondylar ridge (aF_LIR) were considered for comparative analyses (Table A1). No consistent differences in angular orientation were observed between sexes or sides.

### 3.2. Analysis of the Histological Findings

**Figure 2:**
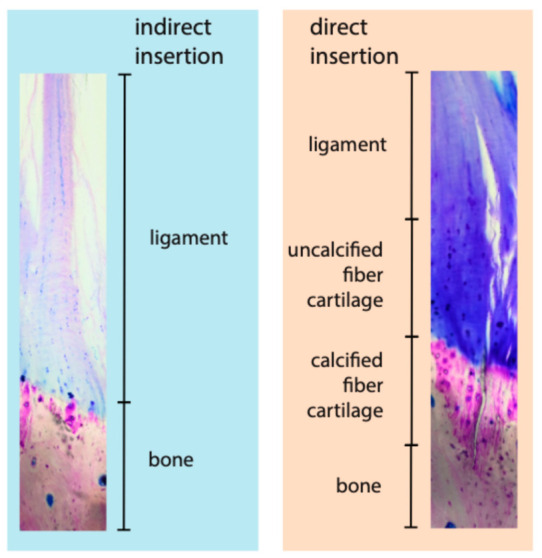
Representative histological images illustrating the structural difference between indirect and direct insertions. The indirect insertion (blue) shows a simplified attachment with ligament fibers inserting directly into bone, whereas the direct insertion (orange) demonstrates the characteristic four-layer enthesis consisting of ligament, uncalcified fibrocartilage, calcified fibrocartilage, and bone.

Histological analysis of the femoral insertion sites revealed a consistent pattern for both the ACL and PCL. Centrally, a four-layered direct insertion was identified (see Figure 2), consisting of ligament tissue, non-calcified fibrocartilage, calcified fibrocartilage, and bone. This region was characterized by a gradual transition between tissue types and a serrated interface between calcified fibrocartilage and bone.

**Figure 3:**
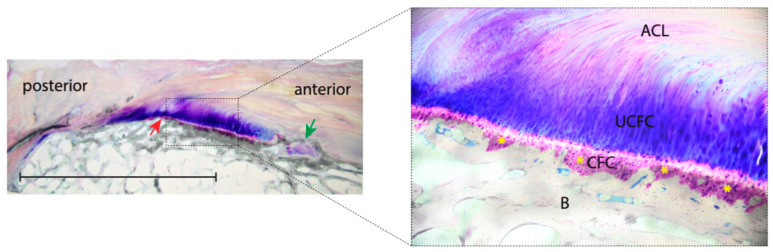
(**Left**): Overview of the femoral ACL enthesis showing the direct insertion region, which is bordered anteriorly by a bony elevation (green arrow). A bony ridge is also present at the posterior margin of the direct insertion (red arrow). Scale bar indicates 10 mm. (**Right**): In the higher-magnification view of the direct ACL insertion, the characteristic four-layer transition is demonstrated: Anterior cruciate ligament (ACL), uncalcified fibrocartilage (UCFC), calcified fibrocartilage (CFC), and bone (B). The interdigitation between calcified fibrocartilage and bone is indicated by asterisks. (Staining: Giemsa; undecalcified thin-ground section.).

Surrounding this central area, a two-layered indirect insertion was observed (see Figure 2), in which collagen fibers inserted directly into bone without a fibrocartilaginous transition. The extent and distribution of the indirect insertion varied between specimens but generally extended toward the articular cartilage. Localized ridge-like osseous elevations were observed at the margins of the direct insertion, particularly at the anterior and posterior boundaries. Histological analysis identified these structures as the LIR and MIR, consistently located at the edges of the ACL and PCL insertion zones (see Figure 3), supporting their role as anatomical boundaries of the functionally relevant attachment sites. In contrast, the LBR and MBR could not be assessed due to the section orientation, as these structures were oriented parallel to the section plane. Therefore, histological support for ridge-like elevations at the margins of the direct insertion was limited to the intercondylar ridges and could not be extended to the bifurcate ridges.

**Figure 4:**
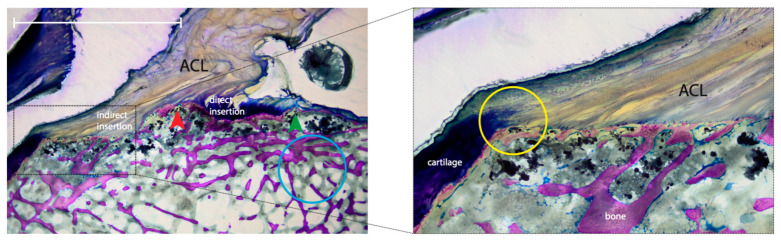
(**Left**): Low-magnification overview showing the distinct morphology of the direct insertion and the indirect insertion region of the ACL. Bony elevations are visible at the anterior margin (green arrow) and the posterior margin (red arrow) of the direct insertion. The blue circle marks an area in which trabecular bone is oriented radially toward the direct insertion. Scale bar indicates 10 mm. (**Right**): Higher-magnification view of the indirect insertion: the indirect attachment of the ACL to bone extends toward the dorsal articular cartilage of the lateral femoral condyle. In this region, some ACL fibers insert directly into the cartilage (yellow circle).

**Figure 5:**
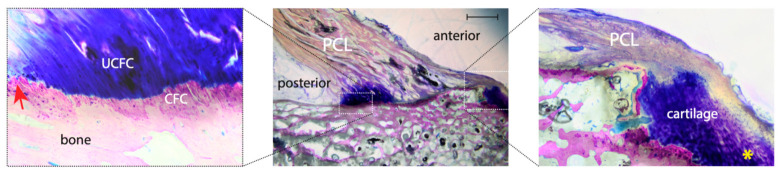
(**Middle**): Overview demonstrating the close relationship of the PCL to the anterior margin of the intercondylar roof and to the anterior articular cartilage of the medial femoral condyle. Scale bar indicates 10 mm. (**Left**): Higher-magnification view of the direct PCL insertion showing a multilayered enthesis with uncalcified fibrocartilage (UFC), calcified fibrocartilage (CFC), and bone (B) of the distal femur. The interface between calcified fibrocartilage and bone exhibits a saw-tooth morphology. A bony elevation that proximally delineates the direct insertion is indicated by the red arrow. (**Right**): Detail view of the anterior rim of the intercondylar fossa demonstrating transition of PCL fibers into articular cartilage; the asterisk marks this region.

Additionally, fibers of the ACL as well as the PCL have been shown to insert not only into the bone via direct or indirect insertion, but also into adjacent cartilage as seen in Figure 4 for the ACL and Figure 5 for the PCL.

### 3.3. Comparison of Histological and CT Findings

For the descriptive comparison of CT-based and histological ridge assessment, the frequencies of the respective ridges were recorded (Table 2). Specimens lacking cruciate ligament insertions on the lateral (n = 3) or medial (n = 2) wall of the intercondylar fossa were excluded from the method comparison. Comparison of detection rates between the two approaches showed minor differences. Histological identification of the LIR was higher than CT detection when using overall CT prevalence as a reference (+9.1 percentage points), whereas histological identification of the MIR was slightly lower than CT detection (−2.2 percentage points). However, because histological denominators differed from CT denominators and no quantitative CT–histology co-registration was performed, these comparisons are descriptive only.

## 4. Discussion

Quantitative 3D-CT assessment identified a high prevalence of the lateral and medial intercondylar ridges (LIR 85%, MIR 80%), contrasted by a low prevalence of the lateral and medial bifurcate ridges (LBR 25%, MBR 10%) in this anatomical cohort. In addition, ridge identification was reproducible between observers, with Cohen’s κ values in the substantial-to-almost-perfect range. Histologically, both ACL and PCL femoral insertions demonstrated a consistent central “direct” insertion with a four-layer transition (ligament–non-calcified fibrocartilage–calcified fibrocartilage–bone), surrounded by an “indirect” insertion with collagen fibers inserting directly into bone. Ridge-like osseous elevations were observed at the margins of the direct insertion, most consistently at anterior and posterior boundaries anatomically consistent with the expected locations of the LIR and MIR. This supports the interpretation of these ridges as potential anatomical boundary markers of the direct insertion, while acknowledging that no quantitative CT–histology co-registration was performed.

Taken together, these findings support a pragmatic “landmark hierarchy”: LIR and MIR appear to be high-yield landmarks that are frequently present and reliably identifiable, whereas LBR and MBR are low-yield landmarks that may refine localization when present but cannot be depended upon routinely. This interpretation aligns with contemporary emphasis on reproducible, anatomy-based tunnel positioning in anatomic reconstruction and with the concept that the functionally dominant insertion is likely concentrated within the direct insertion region rather than the entire macroscopic footprint [1]. The histological observation of a layered enthesis at the direct insertion provides a plausible biological substrate for “functional attachment”, consistent with modern enthesis mechanobiology demonstrating tissue specialization and graded interfaces for force transmission [24]. In that framework, ridge-like elevations adjacent to the direct insertion may reflect local adaptation at the interface where load transfer is highest, rather than merely incidental surface irregularities.

The high prevalence of LIR and MIR in our cohort is consistent with their established use as landmarks within the intercondylar fossa and with descriptions of these ridges as boundaries of cruciate ligament insertions [15]. In contrast, the lower prevalence of bifurcate ridges (LBR/MBR) supports previous observations that these structures are more variably expressed and may be difficult to identify due to individual anatomy, imaging conditions, or degenerative remodeling [15,17]. This variability likely contributes to inconsistent ridge identification and tunnel placement, as prior 3D model studies have shown that even experienced surgeons may differ in their interpretation of femoral ridges under standardized conditions [18]. Our findings refine this issue by distinguishing commonly detectable intercondylar ridges (LIR/MIR) from less consistently observed bifurcate ridges (LBR/MBR), while providing histological evidence that the more consistent ridges correspond to the border of the direct insertion. Together, these observations support the use of complementary cartilage-based and imaging-based reference strategies to improve the reproducibility of femoral footprint localization.

The present findings should be interpreted in light of age-related changes in ACL insertion morphology. Previous studies have reported alterations in femoral ACL footprint shape with aging, including a shift toward smaller, ribbon-like configurations [19]. Because our specimens originated from elderly donors, cortical remodeling, cartilage degeneration, notch narrowing, and osteophyte formation may have influenced both ridge visibility and insertion-site morphology, potentially contributing to the lower detectability of subtle structures such as bifurcate ridges. This is consistent with reports that intercondylar notch morphology varies with degenerative change and ACL pathology [14]. Consequently, the prevalence values observed in this cohort may not be directly representative of the younger, more physically active population typically undergoing ACL reconstruction, limiting the external validity of our findings.

On the imaging side, our finding of substantial interobserver agreement supports the increasing use of cross-sectional imaging for the objective assessment of femoral tunnel position and notch wall morphology [2]. Our results also integrate well with the concept that local bone characteristics may encode functional attachment: CT-based density mapping has demonstrated consistent high-density regions on the lateral intercondylar wall with spatial correspondence to reported ACL attachment centers, interpreted as potential indicators of the direct insertion zone [23]. Finally, while most surgical landmark work focuses on ACL anatomy, our combined CT–histology evaluation contributes to the comparatively smaller body of work on PCL femoral insertion, which also demonstrates regionally specialized insertion patterns and functionally distinct fiber areas [27,28,29].

Clinically, femoral tunnel malposition remains a major modifiable cause of suboptimal outcomes and revision after ACL reconstruction [3]. Given the documented variability of tunnel placement despite structured landmark-based techniques [4,5], our findings support a hierarchical approach to femoral footprint orientation that prioritizes the consistently identifiable LIR (ACL side) and MIR (PCL side) as primary landmarks, while treating bifurcate ridges as optional secondary references. Such a strategy may improve standardization and training by reducing reliance on inconsistent or absent landmarks. However, as the present study did not evaluate trainee performance, tunnel accuracy, or clinical outcomes, these educational and clinical implications remain hypothesis-generating and require validation in anatomical, simulation, and clinical studies.

These findings may inform adjunctive image-guided strategies, but only as anatomical rationale. When ridge morphology is ambiguous, cartilage-based landmarks, fluoroscopy, navigation, or patient-specific guides may represent complementary approaches. However, the present study did not evaluate these techniques or assess tunnel-placement accuracy. Future anatomical studies evaluating specifically the drilling process and clinical imaging studies are required to determine whether CT-visible ridge patterns improve femoral tunnel localization.

Scientifically, the combined CT–histology approach strengthens the inference that macroscopic osseous morphology relates to insertion microanatomy. The observed layered structure of the direct insertion and the ridge-like elevations at its border are congruent with the concept that entheses are specialized for load transfer and may exhibit structural adaptation at the mineralized interface [24]. Complementary clinical data showing altered mineralized tissue states at the femoral ACL enthesis after failure further support that the insertion is a dynamic structure responsive to loading and injury [25]. Animal model work additionally suggests that enthesis deformation behavior is load-dependent, reinforcing that “functional attachment” is not purely geometric but also biomechanical [30].

A key strength of this study is the integration of 3D-CT-based landmark assessment with histologic characterization of cruciate ligament insertions, allowing interpretation of ridge morphology in relation to microanatomic insertion structure rather than relying solely on surface anatomy. The standardized CT reorientation and the substantial-to-almost-perfect interobserver agreement support the reproducibility of ridge identification under the applied protocol. In addition, our study evaluates both ACL- and PCL-related ridges, addressing a gap where PCL landmarks are often less systematically assessed than ACL landmarks.

The clinical implications of these findings should be interpreted cautiously. The present study demonstrates anatomical variability and histological relationships of femoral cruciate ligament attachment sites, but it did not assess tunnel drilling, intraoperative landmark recognition, graft biomechanics, postoperative tunnel position, or patient outcomes. Therefore, the data do not prove that prioritizing LIR or MIR improves tunnel-placement accuracy or surgical results. Rather, they provide an anatomical rationale for future validation studies.

Several limitations should be acknowledged. First, the small sample size limits statistical power to detect subtle effects of sex, side, or age and restricts more complex modeling. Second, the elderly donor cohort represents a major limitation and restricts generalizability to the typical ACL reconstruction population. The specimens originated predominantly from older individuals, whereas ACL reconstruction is commonly performed in younger and physically active patients. Age-related remodeling of the intercondylar notch, cortical surface, cartilage interface, and cruciate ligament footprint may have influenced ridge detectability and insertion morphology. Third, left and right femora from the same donor were analyzed descriptively as separate specimens, which may introduce within-subject dependence and overestimate the precision of subgroup comparisons. Future studies with larger cohorts should use statistical models accounting for donor-level clustering. Fourth, no quantitative CT–histology co-registration was performed; therefore, the relationship between CT-visible ridges and histological insertion morphology should be interpreted as descriptive anatomical association rather than direct spatial correspondence. Fifth, histological section orientation did not allow direct assessment of the bifurcate ridges, limiting conclusions regarding LBR and MBR to CT-based detectability and interobserver agreement. Their relationship to direct or indirect insertion zones could not be histologically validated in the present study. Finally, ridge detection on 3D-CT is influenced by image resolution, volume-rendering parameters, segmentation, and observer interpretation; methodological differences across studies may therefore contribute to variability in reported ridge prevalence.

Future research should (i) replicate these analyses in larger and younger cohorts and in knees with documented ACL deficiency or osteoarthritic change to quantify how remodeling influences ridge visibility and insertion morphology; (ii) use statistical models that account for paired specimens and donor-level clustering; (iii) combine high-resolution CT or micro-CT with serial histological sectioning, fiducial markers, or three-dimensional histological reconstruction to enable quantitative CT–histology co-registration; (iv) include additional orthogonal or serial histological sectioning planes specifically designed to capture bifurcate ridges; and (v) link ridge morphology and direct insertion boundaries to actual tunnel-placement accuracy in cadaveric drilling experiments or clinical postoperative imaging studies.

## 5. Conclusions

In this combined CT–histology study of femoral cruciate ligament attachment sites, LIR and MIR were frequently present and reproducibly identifiable, whereas bifurcate ridges were uncommon. Histology demonstrated a consistent direct insertion structure for both ACL and PCL, with ridge-like elevations at the margins of the direct insertion, supporting LIR and MIR as anatomical boundaries of functionally relevant attachment. These findings support prioritizing LIR and MIR as primary osseous landmarks for femoral attachment orientation, while treating bifurcate ridges as adjunctive landmarks when clearly present, and they provide a microanatomic rationale for integrating surface morphology with functional insertion concepts in anatomic reconstruction. From a precision orthopedics perspective, preoperative or postoperative 3D-CT characterization of ridge patterns and subcortical bone signatures may provide imaging biomarkers to support individualized tunnel planning and selection of adjunct guidance when ridge morphology is equivocal.

## Figures and Tables

**Table 1:** CT-based ridge prevalence according to sex and side, including exploratory group differences and interobserver agreement.

Ridge	Total n/N (%) [95% CI]	Female n/N (%)	Male n/N (%)	Difference Female−Male, pp [95% CI]	*p*-Value Sex	Left n/N (%)	Right n/N (%)	Difference Left−Right, pp [95% CI]	*p*-Value Side	Cohen’s κ
LIR	17/20 (85.0) [64.0 to 94.8]	12/14 (85.7)	5/6 (83.3)	+2.4 [−26.7 to 43.4]	0.891	7/10 (70.0)	10/10 (100.0)	−30.0 [−60.3 to 3.8]	0.060	0.773
LBR	5/20 (25.0) [11.2 to 46.9]	3/14 (21.4)	2/6 (33.3)	−11.9 [−51.1 to 23.4]	0.573	2/10 (20.0)	3/10 (30.0)	−10.0 [−43.5 to 26.5]	0.606	0.894
MIR	16/20 (80.0) [58.4 to 91.9]	12/14 (85.7)	4/6 (66.7)	+19.0 [−15.8 to 57.1]	0.329	7/10 (70.0)	9/10 (90.0)	−20.0 [−51.4 to 16.0]	0.264	0.773
MBR	2/20 (10.0) [2.8 to 30.1]	2/14 (14.3)	0/6 (0.0)	+14.3 [−26.1 to 39.9]	0.329	1/10 (10.0)	1/10 (10.0)	0.0 [−31.5 to 31.5]	1.000	0.828

Values are presented as n/N and percentage. Differences are reported as absolute percentage-point differences with 95% confidence intervals. Positive values indicate higher prevalence in females or left-sided specimens, respectively. *p*-values refer to exploratory group comparisons based on the statistical approach used in the original analysis. CI, confidence interval; pp, percentage points; LIR, lateral intercondylar ridge; LBR, lateral bifurcate ridge; MIR, medial intercondylar ridge; MBR, medial bifurcate ridge; κ, Cohen’s kappa.

**Table 2:** Comparison of CT-based ridge detection and histological assessment.

Ridge	CT Assessment n/N (%)	Histologically Evaluable Specimens	Histological Assessment n/N (%)	Difference Histology − CT, pp	Interpretation
LIR	17/20 (85.0)	17	16/17 (94.1)	+9.1	Histologically identified at the margin of the direct ACL insertion
MIR	16/20 (80.0)	18	14/18 (77.8)	−2.2	Histologically identified at the margin of the direct PCL insertion
LBR	5/20 (25.0)	Not assessable	Not assessable	-	Not histologically assessable because of section orientation
MBR	2/20 (10.0)	Not assessable	Not assessable	-	Not histologically assessable because of section orientation

Values are presented as n/N and percentage. CT assessment refers to all 20 specimens. Histological assessment was limited to evaluable specimens; specimens lacking cruciate ligament insertions on the lateral wall (n = 3) or medial wall (n = 2) were excluded. Difference was calculated as histological percentage minus CT percentage and is reported in percentage points. Bifurcate ridges were not histologically assessable because of section orientation. CT, computed tomography; pp, percentage points; LIR, lateral intercondylar ridge; MIR, medial intercondylar ridge; LBR, lateral bifurcate ridge; MBR, medial bifurcate ridge.

## Data Availability

The original contributions presented in this study are included in the article. Further inquiries can be directed to the corresponding author.

## Abbreviations

The following abbreviations are used in this manuscript:

aF	Femoral axis
LIR	Lateral intercondylar ridge axis
LBR	Lateral bifurcate ridge axis
MIR	Medial intercondylar ridge axis
MBR	Medial bifurcate ridge axis
aF–LIR	Angle between femoral axis and lateral intercondylar ridge
aF–LBR	Angle between femoral axis and lateral bifurcate ridge
aF–MIR	Angle between femoral axis and medial intercondylar ridge
aF–MBR	Angle between femoral axis and medial bifurcate ridge
LIR–LBR	Angle between lateral intercondylar ridge and lateral bifurcate ridge
MIR–MBR	Angle between medial intercondylar ridge and medial bifurcate ridge
3D-CT	three-dimensional computed tomography
ACL	anterior cruciate ligament
ACLR	anterior cruciate ligament reconstruction
ADC	apex of the deep cartilage
MMA	methyl methacrylate
MRI	magnetic resonance imaging
PCL	posterior cruciate ligament

## Appendix A

**Table A1:** Summary of CT-scan acquired angular measurements (central tendency and variance) between ridge and aF, or between two ridges. Missing values were denoted using (-).

Angle [°]		Mean	SD	Min	Max	Range
aF–LIR	Female (n = 12)	132.76	5.83	120.0	141.2	21.2
	Male (n = 5)	129.28	11.57	119.4	146.1	26.7
	Left (n = 7)	127.92	7.19	119.4	138.4	19.0
	Right (n = 10)	134.41	7.22	120.5	146.1	25.7
	Total (n = 17)	131.74	7.72	119.4	146.1	26.7
aF–LBR	Female (n = 3)	58.92	12.95	50.3	73.8	23.5
	Male (n = 2)	52.85	16.67	41.1	64.6	23.6
	Left (n = 2)	63.22	14.98	52.6	73.8	21.2
	Right (n = 3)	52.00	11.88	41.1	64.6	23.6
	Total (n = 5)	56.49	12.82	41.1	73.8	32.8
LIR–LBR	Female (n = 3)	101.35	13.38	87.6	114.3	26.7
	Male (n = 2)	86.98	9.01	80.6	93.3	12.7
	Left (n = 2)	100.94	18.90	87.6	114.3	26.7
	Right (n = 3)	92.04	10.85	80.6	102.2	21.6
	Total (n = 5)	95.60	13.11	80.6	114.3	33.7
aF–MIR	Female (n = 12)	73.06	7.68	61.9	87.7	25.8
	Male (n = 5)	66.83	4.07	61.4	70.5	9.1
	Left (n = 7)	69.75	4.80	61.9	76.4	14.6
	Right (n = 9)	72.86	8.94	61.4	87.7	26.3
	Total (n = 17)	71.50	7.37	61.4	87.7	26.3
aF–MBR	Female (n = 2)	141.22	8.53	135.2	147.3	12.1
	Male (n = 0)	-	-	-	-	-
	Left (n = 1)	135.19 *	-	-	-	-
	Right (n = 1)	147.26 *	-	-	-	-
	Total (n = 2)	141.22	8.53	135.2	147.3	12.1
MIR–MBR	Female (n = 2)	80.22	15.19	69.5	91.0	21.5
	Male (n = 0)	-	-	-	-	-
	Left (n = 1)	90.96 *	-	-	-	-
	Right (n = 1)	69.48 *	-	-	-	-
	Total (n = 2)	80.22	15.19	69.5	91.0	21.5

* No mean as only one data point.
